# Implementing Mailed Colorectal Cancer Fecal Screening Tests in Real-World Primary Care Settings: Promising Implementation Practices and Opportunities for Improvement

**DOI:** 10.1007/s11121-023-01496-3

**Published:** 2023-03-23

**Authors:** Sarah D. Hohl, Annette E. Maxwell, Krishna P. Sharma, Juzhong Sun, Thuy T. Vu, Amy DeGroff, Cam Escoffery, Dara Schlueter, Peggy A. Hannon

**Affiliations:** 1 Health Promotion Research Center, University of Washington, Seattle, WA USA; 2 Office of Community Health, Department of Family Medicine and Community Health, Madison, WI USA; 3 Fielding School of Public Health, University of California Los Angeles, Los Angeles, CA USA; 4 Totally Joined for Achieving Collaborative Techniques, Atlanta, GA USA; 5 Division of Cancer Prevention and Control, National Center for Chronic Disease Prevention and Health Promotion, CDC, Atlanta, GA USA; 6 Rollins School of Public Health, Emory University, Atlanta, GA USA

**Keywords:** Mailed fecal testing implementation, Evidence-based interventions (EBIs), Colorectal cancer (CRC) screening, Safety-net clinics, Fecal immunochemical testing, Fecal occult blood test, Mailed outreach, Colorectal neoplasms

## Abstract

Colorectal cancer (CRC) screening reduces morbidity and mortality, but screening rates in the USA remain suboptimal. The Colorectal Cancer Control Program (CRCCP) was established in 2009 to increase screening among groups disproportionately affected. The CRCCP utilizes implementation science to support health system change as a strategy to reduce disparities in CRC screening by directing resources to primary care clinics to implement evidence-based interventions (EBIs) proven to increase CRC screening. As COVID-19 continues to impede in-person healthcare visits and compel the unpredictable redirection of clinic priorities, understanding clinics’ adoption and implementation of EBIs into routine care is crucial. Mailed fecal testing is an evidence-based screening approach that offers an alternative to in-person screening tests and represents a promising approach to reduce CRC screening disparities. However, little is known about how mailed fecal testing is implemented in real-world settings. In this retrospective, cross-sectional analysis, we assessed practices around mailed fecal testing implementation in 185 clinics across 62 US health systems. We sought to (1) determine whether clinics that do and do not implement mailed fecal testing differ with respect to characteristics (e.g., type, location, and proportion of uninsured patients) and (2) identify implementation practices among clinics that offer mailed fecal testing. Our findings revealed that over half (58%) of clinics implemented mailed fecal testing. These clinics were more likely to have a CRC screening policy than clinics that did not implement mailed fecal testing (*p* = 0.007) and to serve a larger patient population (*p* = 0.004), but less likely to have a large proportion of uninsured patients (*p* = 0.01). Clinics that implemented mailed fecal testing offered it in combination with EBIs, including patient reminders (92%), provider reminders (94%), and other activities to reduce structural barriers (95%). However, fewer clinics reported having the leadership support (58%) or funding stability (29%) to sustain mailed fecal testing. Mailed fecal testing was widely implemented alongside other EBIs in primary care clinics participating in the CRCCP, but multiple opportunities for enhancing its implementation exist. These include increasing the proportion of community health centers/federally qualified health centers offering mailed screening; increasing the proportion that provide pre-paid return mail supplies with the screening kit; increasing the proportion of clinics monitoring both screening kit distribution and return; ensuring patients with abnormal tests can obtain colonoscopy; and increasing sustainability planning and support.

## Introduction

Colorectal cancer (CRC) screening by various modalities, including colonoscopy and stool-based tests, reduces morbidity and mortality (Issaka et al., 2019; Lieberman et al., 2000). The United States Preventive Services Task Force currently recommends CRC screening among average-risk, asymptomatic adults ages 45–75 (US Preventive Services Task Force et al., 2021) but screening rates remain suboptimal. The National Colorectal Cancer Roundtable’s 80% in Every Community initiative builds on previous efforts to achieve 80% CRC screening test use among eligible adults by 2018 (National Colorectal Cancer Roundtable, 2020). However, by the end of 2018, only 46% of patients at Federally Qualified Health Center (FQHC) clinics—compared to 62% of all eligible people—were up to date with CRC screening (Office of Disease Prevention and Health Promotion, n.d.; Muthukrishnan et al., 2019). These data underscore a need to address persistent screening disparities experienced by FQHC patient populations, who overwhelmingly and increasingly include patients with incomes below the federal poverty level, patients who are uninsured or insured by Medicaid, racial and ethnic minority groups, and those living in rural areas (Nath et al., 2016).

In 2009, the Centers for Disease Control and Prevention (CDC) initiated the first of three sequential multi-year cooperative agreements of the Colorectal Cancer Control Program (CRCCP) (Joseph et al., 2011). In 2015, CDC revised the CRCCP model to utilize implementation science to support health system change to improve and increase CRC screening through implementation of evidence-based interventions. This approach supports CDC’s strategy to reduce disparities in CRC screening by directing resources to primary care clinics that serve patient populations with historically low screening rates, such as FQHC clinics (Joseph, 2016; Joseph et al., 2011; Muthukrishnan et al., 2019). The current 5-year CRCCP initiative (DP20-2002) was funded in 2020 and includes 35 CRCCP recipients including state health departments, universities, tribal, and other organizations. The Community Preventive Services Task Force (CPSTF) recommends multiple evidence-based interventions (EBIs) to increase CRC screening in the Community Guide (https://www.thecommunityguide.org). CDC requires that CRCCP recipients implement at least two of the following four EBIs: patient/client reminders, provider reminders, provider assessment and feedback, and reducing structural barriers at the patient, provider, and system levels (e.g., expanded clinics hours, child/elder care, mailed fecal testing) (Community Preventive Services Task Force, 2015; Sabatino et al., 2012). These EBIs are described in Table 1.

**Table 1:** Evidence-based interventions prioritized by the CDC Colorectal Cancer Control Program

EBI	Definition
Patient or client reminders	Written (letter, postcard, email, or text message) or telephone messages (including recorded or automated messages) advising patients that they are due for screening. Patient reminders can be general to reach a group of people or tailored to reach one person.
Provider reminders	Reminders inform health care providers that a patient is due or past due for a cancer screening test. A recall is another form of provider reminder that alerts providers that a client is overdue for screening. The reminders can be provided in different ways, such as in patient charts or by e-mail.
Provider assessment and feedback	Interventions that evaluate provider performance in delivering or offering screening to patients are called assessments. Presentation of information to providers about their performance in providing screening services is called feedback.
Reducing structural barriers	Strategies implemented to reduce structural barriers, i.e., non-economic burdens or obstacles—such as inconvenient clinic hours or lack of transportation—that make it difficult for people to access cancer screening.

Offering fecal CRC screening is an effective strategy to reduce structural barriers faced by patient populations with the lowest CRC screening rates (Gupta et al., 2013, 2020). Evidence-based fecal tests include fecal DNA tests, guaiac fecal occult blood test (FOBT), and fecal immunochemical tests (FIT), the latter of which is preferred given its greater adherence, sensitivity, and associations with higher decreases in CRC mortality compared to FOBT (Issaka et al., 2019; Lee et al., 2014; Levin et al., 2018). Mailed fecal testing, in which eligible patients receive, complete, and return FOBT/FIT tests by mail, is a cost-effective, evidence- and population-based approach to increasing CRC screening rates and reducing health disparities (Gupta et al., 2020; Issaka et al., 2019; Jager et al., 2019; Selby et al., 2022). This approach mitigates barriers to CRC screening that many patients face related to transportation to the clinic, limited clinic hours, and need for child or elder care and addresses many patients’ desire for a less-invasive screening test (Gupta et al., 2013; Lee et al., 2021; Mehta et al., 2016). Implementing mailed fecal testing has resulted in increased screening rates across multiple safety-net clinic settings (Joseph, 2016; Lee et al., 2021; Murphy et al., 2021; Singal et al., 2016). Although much evidence supports the use of mailed fecal testing as an effective approach for bolstering screening rates among disproportionately affected populations (Joseph, 2016; Mehta et al., 2016; Murphy et al., 2021; Singal et al., 2016), it remains underutilized in practice (Issaka et al., 2019; Wang et al., 2021). When combined with EBIs supported by CRCCP, mailed fecal testing may have even greater potential to increase screening and reduce disparities in CRC incidence and mortality (Davis et al., 2018).

As COVID-19 continues to impede in-person healthcare visits, mailed fecal testing offers an evidence-based alternative to in-person screening tests, and represent an especially promising approach to reduce CRC screening disparities. Although the effectiveness and efficacy of mailed fecal testing has been established, little is known about how mailed fecal testing is implemented in real-world settings that primarily serve patient populations with historically low screening rates who also experience high CRC morbidity and mortality burden, including within clinics funded to implement EBIs. Even less is known about how mailed fecal testing has been implemented during a public health emergency, like the COVID-19 pandemic. In this study, we assessed practices around mailed fecal testing and EBI implementation in clinics across 62 US health systems. This analysis can assist in identifying promising practices and implementation gaps of mailed fecal testing implementation in clinics and health systems and potential strategies to optimize CRC screening and reduce screening disparities during and beyond the COVID-19 pandemic.

## Methods

### Data Collection

In this retrospective, cross-sectional analysis, we utilized data from a web-based survey of CRCCP clinics participating in the CRCCP in 2020 as well as data routinely collected by CDC from all participating clinics in 2015, the baseline year for the 2015–2020 CRCCP funding cycle (DP15-2015).

#### CRCCP Survey

In June 2019, CDC partnered with the University of Washington (UW), the University of California at Los Angeles, and Emory University to develop a web-based survey instrument to assess how CRCCP clinics implemented EBIs. The team conducted a literature review to identify relevant existing measures of constructs of interest. The draft survey was pilot tested between December 2019 and January 2020 by nine health professionals familiar with the CRCCP, including CRCCP award recipients and CDC staff. Feedback from pilot testing was integrated to refine questions as needed; ensure accurate programming and skip patterns; and establish the estimated time required to complete the survey. The final survey included 24 main questions and took approximately 20 min to complete. The survey was approved by the office of management and Budget (OMB, #0920-0879, exp. 01/31/2021) and was categorized as program evaluation and granted exemption by the University of Washington Institutional Review Board. It is available as supplementary material.

To characterize EBI implementation and mailed fecal testing practices, we selected three domains from the full survey for the current analysis: (1) EBI implementation practices, (2) mailed fecal testing management and health information technology (IT) practices, and (3) sustainability and unintended consequences. EBI implementation practices included dichotomous items to assess whether the four EBIs (i.e., patient/client reminders, provider reminders, provider assessment and feedback, and reducing structural barriers) were implemented at each clinic, and specific strategies used to implement each intervention. Mailed fecal testing management items assessed whether mailed fecal testing was managed at the health system or clinic level. Health IT items, defined as electronic systems that health care professionals and patients use to store, share, and analyze health information, (e.g., electronic health records [EHR], electronic prescribing, and patient-provider communication via an online portal) (U.S. Department of Health and Human Services, 2021), assessed how clinics used these systems to support implementation of mailed fecal testing. For sustainability of EBI implementation, respondents were asked to report the extent to which their clinics had leadership support, funding stability, organizational capacity, and program adaptation in place, on a scale of 1 (not at all) to 5 (to a very great extent). Definitions for each were provided in the survey based on the Program Sustainability Assessment Tool (PSAT) (Luke et al., 2014) and are outlined in Table 2. Unintended consequences included a single question regarding the ability of patients with positive fecal tests to get a follow-up colonoscopy.

**Table 2:** Sustainability domains: definitions (Luke et al., 2014)

Domain	Definition
Leadership support	Internal and external environments that support EBI implementation.
Funding stability	A consistent financial base for implementing EBIs.
Organizational capacity	The necessary support and buy-in from clinic staff to effectively manage EBIs and supporting activities.
Program adaption	The ability to take actions that adapt EBIs to ensure their ongoing effectiveness.

*EBI* evidence-based intervention

In November 2020, the UW fielded the assessment to contacts at 303 partner clinics across 15 CRCCP recipients via a one-time web-based survey using Research Electronic Data Capture (REDCap). These clinics represented 107 health systems. The survey was administered approximately 9 months following the first detected case of COVID-19 in the USA (February 2020) and 4 months after the start of a new 5-year CRCCP funding cycle (July 2020). The timeline for administering the assessment coincided with the outbreak of COVID-19 in the USA and the transition between two CRCCP funding cycles. Although we had originally planned to survey partners of award recipients in the 2015–2022 CRCCP cycle, given the tremendous financial and human resources strains on health systems during that time (Krist et al., 2020), we postponed administration of the survey. Thus, clinics that were invited to participate in the voluntary assessment were partners of award recipients who had received funding in two CRCCP cycles (2015–2020 and 2020–2025). Participants were given up to 6 weeks to complete the survey. Reminders were sent to non-responders, and UW staff conducted follow-up with assistance from CDC staff as needed.

#### Clinic Data

**Fig. 1:**
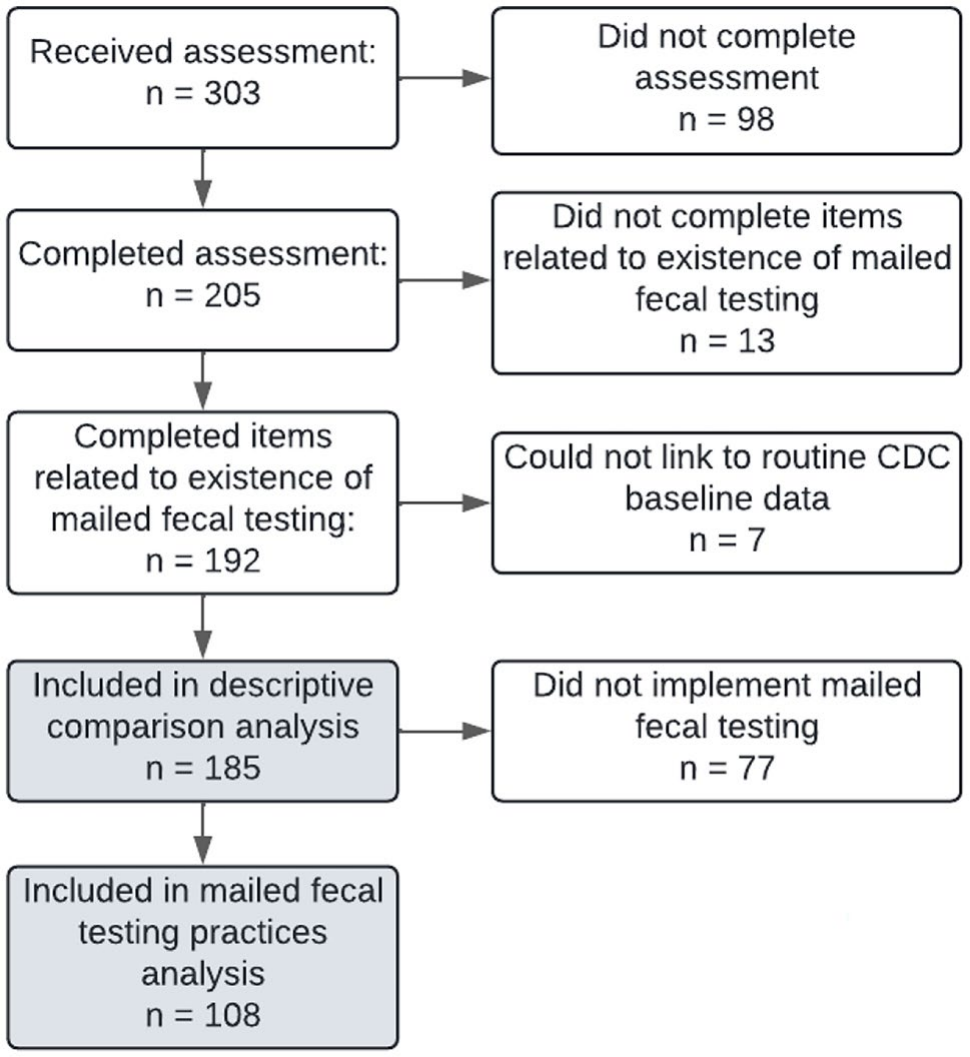
Selection of partner clinics for analysis

We linked assessment data with clinic data collected by CDC during routine CRCCP evaluation activities at baseline (in 2015). Baseline clinic data included clinic characteristics not collected as part of the assessment (Satsangi & DeGroff, 2016). Clinics were included in the current analysis if they (1) completed the CRCCP survey in 2020 and answered questions regarding existence of mailed fecal testing and (2) submitted routine evaluation data at baseline. Figure 1 illustrates the process of clinic selection for inclusion in the analysis.

#### Data Analysis

Data were analyzed using SAS software, version 9.4 (SAS Institute Inc.). We calculated summary statistics and conducted chi-square tests to compare clinics that did and did not implement mailed fecal testing with respect to clinic type, location, proportion of uninsured patients, clinic size, number of providers, primary test type, and having a CRC screening champion and a CRC screening policy. To summarize practices of clinics that implemented mailed fecal testing (*n* = 108), we calculated and present descriptive statistics for EBI implementation, mailed fecal testing management and health IT practices, and factors related to sustainability for those clinics. For the four items related to sustainability, we combined the responses “small extent” with “moderate extent” and the responses “great extent” with “very great extent.”

## Results

### Characteristics of Clinics That Did and Did Not Implement Mailed Fecal Testing

A total of 185 clinics representing 62 health systems across 15 states were included in the descriptive comparison analysis. This number represents 61% of all clinics that received the survey (Fig. 1). Of these 185 clinics, 25 (14%) represented the only clinic in their health system whereas 160 (86%) were one of multiple clinics within a single health system included in the analysis (Table 3); 108 (58%) implemented mailed fecal testing and 77 (42%) did not (Table 4). Most clinics across the entire sample were community health centers (CHCs)/FQHCs, located in metro areas (as defined by the U.S. Department of Agriculture classifications), had fewer than 5 providers, and had screening champions. Clinics that did and did not implement mailed fecal testing did not significantly differ with respect to these four characteristics. More than half of all clinics (57%) used fecal testing kits as their primary test, 30% of clinics used colonoscopy and, in 11% of clinics, the primary test type varied by provider. Clinics that implemented mailed fecal testing were more likely to use FIT/FOBT kits as their primary test type than clinics that did not implement mailed fecal testing. The latter were marginally more likely to use colonoscopy as their primary test type (*p* = 0.053).

**Table 3:** Clinics and health systems included in the analysis by type (*n* = 185)

***All clinics (n*** = ***185)***				
	Clinics (*n* = 185)	Health systems (*n* = 62)	Health system type	Health systems (*n* = 62)
	*n* (%)	*n* (%)		*n* (%)
Health systems with single clinic	25 (13.5)	25 (40.3)	Academic	2 (3.2)
			CHC/FQHC	20 (32.3)
			Hospital	3 (4.8)
Health systems with multiple clinics	160 (86.5)	37 (59.7)	CHC/FQHC	26 (41.9)
			Hospital	4 (6.5)
			Local health department	1 (1.6)
			Tribal	2 (3.2)
			Other	4 (6.5)

***Clinics that implemented mailed fecal testing (n* = *108)***				
	Clinics (*n* = 108)	Health systems (*n* = 42)	Health system type	Health systems (*n* = 42)
	*n* (%)	*n* (%)		*n* (%)
Single clinic in a single health system	18 (16.7)	18 (42.9)	Academic	1 (2.4)
			CHC/FQHC	12 (28.6)
			Hospital	2 (4.8)
			Tribal	1 (2.4)
			Other	2 (4.8)
Multiple clinics within a health system	90 (83.3)	24 (57.1)	CHC/FQHC	18 (42.9)
			Hospital	3 (7.1)
			Local health department	1 (2.4)
			Other	2 (4.8)

*CHC* Community Health Center, *FQHC* Federally Qualified Health Center

**Table 4:** CRCCP clinic characteristics by existence of mailed fecal testing

	Implemented mailed fecal testing		Did not implement mailed fecal testing		*p* for *χ*^2^ test^a^
	*n*	%	*n*	%	
All clinics (*n* = 185)	108	58.4	77	41.6	
Clinic type					0.30
CHC/FQHC (*n* = 147)	88	81.5	59	76.6	
HS/hospital owned (*n* = 19)	8	7.4	11	14.3	
Other (*n* = 19)	12	11.1	7	9.1	
Urban–rural status					0.12
Metro (*n* = 150)	84	77.8	66	85.7	
Urban (*n* = 25)	16	14.8	9	11.7	
Rural (*n* = 5)	5	4.6	0	0.0	
Unknown (*n* = 5)	3	2.8	2	2.6	
% patients uninsured					0.01
< 5% (*n* = 51)	33	30.6	18	23.4	
5–20% (*n* = 67)	43	39.8	24	31.2	
> 20% (*n* = 42)	16	14.8	26	33.8	
Unknown (*n* = 25)	16	14.8	9	11.7	
Number of patients					0.00
≤ 700 (*n* = 65)	35	32.4	30	39.0	
> 700–1700 (*n* = 59)	28	25.9	31	40.3	
> 1700 (*n* = 59)	45	41.7	14	18.2	
Missing (*n* = 2)	0	0.0	2	2.6	
Number of providers					0.48
< 5 (*n* = 91)	56	51.9	35	45.5	
5–9 (*n* = 38)	19	17.6	19	24.7	
≥10 (*n* = 51)	30	27.8	21	27.3	
Missing (*n* = 5)	3	2.8	2	2.6	
Primary test type					0.05
Colonoscopy (*n* = 55)	26	24.1	29	37.7	
FIT/FOBT kits (*n* = 105)	66	61.1	39	50.6	
Varies by provider (*n* = 20)	15	13.9	5	6.5	
Unknown (*n* = 5)	1	0.9	4	5.2	
CRC screening champion(s)					0.19
No (*n* = 39)	26	24.1	13	16.9	
Yes (*n* = 140)	77	71.3	63	81.8	
Unknown (*n* = 6)	5	4.6	1	1.3	
CRC screening policy					0.01
No (*n* = 13)	3	2.8	10	13.0	
Yes (*n* = 170)	104	96.3	66	85.7	
Unknown (*n* = 2)	1	0.9	1	1.3	

*CRCCP* Colorectal Cancer Control Program, *CHC* Community Health Center, *FQHC* Federally Qualified Health Center, *HS* health system,* FIT* fecal immunochemical test, *FOBT* fecal occult blood test

^a^Clinics with “unknown” or “missing” status were excluded in *χ*^2^ tests

Compared to clinics without mailed fecal testing, clinics which implemented mailed fecal testing were more likely to have a screening policy in place (95% vs. 86%, *p* = 0.007) and to serve a larger patient population (> 1700, *p* = 0.004), but less likely to have a large proportion of uninsured patients (> 20% uninsured, *p* = 0.01).

### Practices of Clinics That Implemented Mailed Fecal Testing

#### Evidence-Based Interventions

Among the 108 clinics that implemented mailed fecal testing, 99 (92%) implemented patient reminders, with the most common strategies being by telephone call (86%), in person (78%), by mail (74%), or a combination of strategies (94%) (Table 5). The majority (68%) of clinics issued up to three reminders for patients to complete CRC screening. A total of 101 (94%) clinics implemented provider reminders, most commonly through electronic health record (EHR) pop-up messages (62%) and flagging patient charts (55%). Among the 94 (87%) clinics that implemented provider assessment and feedback, 67% did so on a weekly or monthly basis and 32% did so quarterly or annually. A total of 95% of clinics implemented practices to reduce structural barriers other than mailed fecal testing, most commonly through onsite translation or language interpreters (69%), assistance in scheduling endoscopy screening appointments (67%), pre-paid materials to return completed tests (64%), patient navigation (59%), and transportation to/from clinics or endoscopic centers (58%).

**Table 5:** Details of EBI implementation among clinics implementing mailed fecal testing (*n* = 108)

		*n*	%
Implemented patient reminders^a,b^		99	91.7
By telephone call^c^		85	85.9
In person/at appointment^c^		77	77.8
By mail (letter/postcard)^c^		73	73.7
By online portal notification^c^		36	36.4
By text message^c^		35	35.4
By email^c^		23	23.2
By more than one method^c^		93	93.9
Number of times a patient could receive a reminder			
1–2	9		8.3
3	73		67.6
≥ 4	14		13.0
Missing	12		11.1
Implemented provider reminders^a,b^		101	93.5
EHR pop-up message^c^		63	62.4
Flagged patient chart^c^		55	54.5
Daily or weekly lists generated indicating patients due for screening^c^		49	48.5
Flagged patient room^c^		6	5.9
By more than one method		55	54.5
Implemented provider assessment and feedback^a^		94	87.0
Weekly/monthly^c^		63	67.0
Quarterly/annually		30	31.9
Implemented practices to reduce structural barriers^a,b^		103	95.4
Provided onsite translation or language interpreter^c^		71	68.9
Provided patients with assistance in scheduling appointments for endoscopic screening (e.g., colonoscopy)^c^		69	67.0
Provided pre-paid mail back materials to send completed tests back to clinic/laboratory^c^		66	64.1
Offered patient navigation^c^		61	59.2
Provided patients with transportation to/from clinic and/or endoscopic center, including providing vouchers or payments for transportation^c^		60	58.3
Offered fecal screening in conjunction with other visit (e.g., flu shot)^c^		52	50.5
Expanded clinic hours^c^		46	44.7
Offered weekend clinic hours^c^		32	31.1
Developed methods (e.g., section in EHR) to track patient barriers^c^		31	30.1
Set up alternative screening sites^c^		4	3.9
Provided or connected patients to childcare^c^		2	1.9

^a^Indicates type of evidence-based intervention implemented

^b^Patient reminders, provider reminders, and reducing structural barriers could be implemented in more than one way

^c^Indicates ways in which each evidence-based intervention was implemented

#### Mailed Fecal Testing Management and Health IT

Among over half (53%) of clinics, mailed fecal testing was managed by the health system, whereas for 40%, mailed fecal testing was managed by the clinic (Table 6). Over two-thirds (68%) of clinics had systems in place to monitor FIT/FOBT distribution and return. Most (≥ 75%) utilized health IT to track both fecal and endoscopic CRC screening test results to follow up with patients.

**Table 6:** Management and IT practices among clinics implementing mailed fecal testing (*n* = 108)

	*n*	%
Management of mailed fecal testing		
By both the health system and clinic	6	5.6
By health system only	57	52.8
By clinic only	43	39.8
By neither health system nor clinic	2	1.9
Monitoring mailed fecal testing		
Both the number of FIT/FOBT kits distributed and the number returned	73	67.6
FIT/FOBT distribution only	7	6.5
Return only	6	5.6
Neither distribution nor return	10	9.3
Don’t know	12	11.1
Health Information Technologies		
*Uses health IT to:*		
Track results of FIT/FOBT to follow up with patients with abnormal results	95	88.0
Ensure patients with abnormal or positive FIT/FOBT are referred for colonoscopy	95	88.0
Track distribution and/or return of FIT/FOBT kits	81	75.0
Track colonoscopy and follow-up colonoscopy results	95	88.0

*IT* information technology, *FIT* fecal immunochemical test, *FOBT* fecal occult blood test

#### Sustainability of EBI Implementation and Unintended Consequences

Over half of clinics (58%) reported having leadership support to a great/very great extent to sustain the implementation of EBIs after the CRCCP screening initiative ends, but only 29% reported having a great/very great extent of funding stability in place to do so (Table 7). Just 44% of clinics had organizational capacity, and 47% had program adaptation in place to a great/very great extent to ensure ongoing EBI implementation. Almost half (46%) reported that some patients with positive FIT/FOBT results did not have resources to get a colonoscopy to finish the screening cycle.

**Table 7:** Sustainability of EBI implementation and unintended consequences (*n* = 108)

Sustainability domains	*n*	%
Leadership support		
Not at all	1	0.9
To a small/moderate extent	40	37.0
To a great/very great extent	63	58.3
Missing	4	3.7
Funding stability		
Not at all	8	7.4
To a small/moderate extent	65	60.2
To a great/very great extent	31	28.7
Missing	4	3.7
Organizational capacity		
Not at all	0	0.0
To a small/moderate extent	56	51.9
To a great/very great extent	48	44.4
Missing	4	3.7
Program adaptation		
Not at all	0	0.0
To a small/moderate extent	53	49.1
To a great/very great extent	51	47.2
Missing	4	3.7
Unintended consequences		
Some patients with positive FIT/FOBT result did not have resources to get a colonoscopy to finish the screening cycle.	50	46.3

## Discussion

In this study, we assessed implementation of mailed fecal testing, an effective and efficacious strategy for CRC screening, in a large number of clinics that partnered with the CRCCP. The COVID-19 pandemic has caused decreases for breast, cervical, and CRC screening, and mailed fecal testing may offer a means to help maintain screening during public health emergencies (Fisher-Borne et al., 2021) and support screening among those who prefer at-home testing even outside of such emergencies. Our study adds to the literature by examining the extent to which clinics implement mailed fecal testing along with other EBIs to promote CRC screening and by assessing clinic characteristics associated with implementing mailed fecal testing. We identified several promising implementation practices that align with mailed fecal testing best practices, as well as several important implementation gaps and opportunities for improving CRC screening through mailed fecal testing implementation.

### Promising Implementation Practices Regarding EBI and Mailed Fecal Testing Implementation

Almost 80% of clinics were CHCs and FQHCs that provide care to groups with lower rates of CRC screening and often experience disadvantage, suggesting that the CRCCP is reaching its intended population of focus. Although the sample size was small, all clinics included in the analysis located in rural areas implemented mailed fecal testing. Offering mailed fecal testing may be particularly beneficial in rural areas where health care accessibility can be a significant barrier to screening (Davis et al., 2018).

A summary published in 2020 by a group of CRC screening experts and stakeholders convened at a CDC-sponsored summit offered nine strategies to maximize the effectiveness of mailed fecal test implementation. These strategies include establishing data infrastructure needed to track mailed fecal tests; practicing effective communication with patients about the tests and how to use them; and implementing approaches for following up with patients with abnormal results (Gupta et al., 2020). Clinics in our study used multiple approaches to implement patient and provider reminders, and most clinics reminded patients up to three times to complete CRC screening. Over half of clinics helped patients schedule endoscopy appointments when necessary, provided pre-paid materials to mail completed fecal tests to the laboratory, and provided patient navigation, all approaches demonstrated to enhance CRC screening adherence and among the best practices summarized at the summit (Gupta et al., 2020). Moreover, almost 90% of clinics utilized health IT to track mailed fecal test results, ensure those with abnormal results were referred for endoscopy, and track endoscopy results; strategies also included in the CDC summit summary to enhance mailed fecal testing implementation and reduce CRC incidence (Gupta et al., 2020). Although the best practice summary on mailed fecal testing was released after our survey was administered, this work illustrates the substantial effort of clinics to promote CRC screening to complement and support mailed fecal testing implementation using strategies aligned with best practices.

Mailed fecal testing was significantly more likely to be implemented by large clinics with more than 1700 patients, suggesting the potential of this strategy to increase population-level screening rates. However, the clinics with the largest proportion of uninsured patients (> 20%) were significantly *less* likely to implement mailed fecal testing. It is likely that clinics with a clinic- or health system-level screening policy in place, clinics of larger size, and those with fewer uninsured patients have more capacity to implement mailed fecal testing than those without a policy, of smaller size, and more uninsured patients. Although these findings underscore the importance of having a clinic- or health system-level CRC screening policy in place, a factor associated with increased screening rates (DeGroff et al., 2018), future research could explore clinic capacity as mailed fecal tests have the potential to reduce disparities in CRC screening among uninsured patients who may be largely served by clinics with lower capacity. Clinics that used colonoscopy as their primary screening test were significantly less likely to implement mailed fecal testing. Providers’ belief in the effectiveness of FIT may influence their willingness to emphasize its use (Thompson et al., 2019). Additionally, not all patients have access to colonoscopy. In one study, a sample of primary care physicians identified colonoscopy as a superior screening test, but less than half agreed colonoscopy was available to their patients (Brown et al., 2015). The influence of provider attitudes on mailed fecal test implementation warrants future research, particularly as the use of FIT-DNA increases in use.

An overwhelming majority of clinics that implemented mailed fecal testing also implemented all four EBIs in efforts to bolster screening rates. This finding is encouraging, as studies have shown that implementation of these EBIs leads to increased CRC screening, especially if several EBIs are implemented simultaneously (Sharma et al., 2021). It is conceivable that mailed fecal testing may be particularly effective if EBIs that remind patients and providers are also in place. A recent analysis of CRCCP clinics showed that by 2018, more than 80% of clinics had patient and provider reminder systems in place, which are intervention strategies prioritized by the CRCCP (Maxwell et al., 2022). However, this finding could also be further indicated that clinics that implemented mailed fecal testing had greater capacity than those that did not. Our study provides details of how and how often these EBIs are implemented, processes of interest for clinic managers, and those involved in implementing EBIs. Our work also illustrates that mailed fecal testing is only one of several strategies clinics utilize to promote CRC screening (Maxwell et al., 2022).

### Opportunities for Improving CRC Screening Through Mailed Fecal Testing

CRC screening by mailed fecal testing is a strategy that is part of the EBI “reducing structural barriers,” but not one specifically prioritized by CRCCP or the CPSTF. Although the 58% of clinics that implemented mailed fecal testing suggest that this evidence-based approach is feasible in these settings, efforts are needed to expand mailed fecal testing to other CRCCP clinics. Among clinics that implemented mailed fecal testing, 36% did not provide patients with pre-paid materials to return tests. Although including prepaid materials removes patient-level barriers and is associated with fecal test completion (Coronado et al., 2018), the infrastructure required for coordinating mailed distribution to patients who are overdue for CRC screening and return of completed test kits to the clinic or laboratory is significant and may present barriers for some clinics. Often, health IT is used to track distribution and return, as patients with abnormal test results need to be notified and referred to a follow-up colonoscopy. In our sample, 32% of clinics did not track both distribution and return, suggesting an opportunity to build health IT capacity to establish those practices. Future research is needed to determine feasible, acceptable, and cost-effective approaches to integrating health IT as part of mailed fecal testing orders, returns, and results. Moreover, even though 88% of clinics used health IT to ensure patients with abnormal fecal tests were referred for colonoscopy, at almost half (46%) of clinics, patients with a positive test did not have resources to obtain a colonoscopy. The CRCCP currently provides limited funding for average risk patients who have low incomes or are under- or uninsured to obtain follow-up colonoscopy; however, not all patients in need are eligible to receive this vital service.

The potential for sustainability of EBIs implemented offers another opportunity for improvement. Only 58% of clinics reported having good leadership support to maintain implementation of CRC screening EBIs, including mailed fecal testing. Fewer clinics reported having funding stability, organizational capacity, or the ability to adapt practices to ensure sustainability of EBI implementation. Although the CRCCP and other programs are designed with sustainability as a long-term goal, sustainability is an on-going challenge in clinics with limited resources and changing priorities that are reflected in budget changes. Future research should address the type of support clinics may need to sustain strategies to increase CRC screening and explore potential sources of funding/support beyond CRCCP.

The group of experts convened at the CDC summit summarized data on the cost-effectiveness of mailed fecal testing (Gupta et al., 2020); however, labor costs relating to fecal testing can be substantial (Coury et al., 2022) and total costs per completed test vary considerably (Baldwin et al., 2022; Kemper et al., 2018). Although implementing multi-component interventions alongside mailed fecal testing (e.g., automated reminders, telephone support, patient navigation) can increase screening rates, they also add cost both in terms of the test itself as well as mailing costs, planning and management, contracting with providers, and using health IT to track patients and fecal test results (Green et al., 2013; Subramanian et al., 2017). Our study suggests that CHC and FQHC clinics in the CRCCP were able to overcome the logistical, personnel, and financial challenges for implementing mailed fecal testing found in prior studies. These findings also suggest that the CRCCP supports clinics to establish systems for mailed fecal testing implementation.

### Limitations

This study assessed practices of clinics that were provided both financial and technical support to implement EBIs, including mailed fecal testing, to reduce CRC screening disparities. Clinics outside the CRCCP may implement fewer EBIs and fewer practices that align with published best practices (Gupta et al., 2020), underscoring a need for evaluation of these clinics’ mailed fecal testing implementation practices, and potential support to improve their implementation. The implementation of our evaluation at the time of a global pandemic and a new CRCCP funding cycle resulted in recruitment and participation of fewer clinics than originally planned. Moreover, we did not assess the extent to which COVID may have influenced clinics’ adoption or scale-up of mailed fecal testing. Future research could investigate factors, including the COVID-19 pandemic, that motivate mailed fecal testing implementation.

The overall purpose of the survey instrument we administered among CRCCP partner clinics was to learn more about how they were implementing CPSTF-recommended EBIs. Consequently, survey questions about those EBIs (e.g., how and how often patients received reminders or patient navigation) did not specifically pertain to mailed fecal testing. However, over half (57%) of all clinics reported FIT/FOBT as their primary test type, so it is possible that the clinic practices were designed to support fecal CRC screening, with screening kits either mailed to patients or provided during a clinic visit. Our survey questions did not discern whether clinics mailed FIT or FOBT kits. FIT is preferred as it is more effective at detecting abnormalities, and patients are more likely to adhere to FIT testing than FOBT (Issaka et al., 2019; Lee et al., 2014; Levin et al., 2018). While FOBT requires multiple stool collections, FIT requires just one, performs better, and may be more acceptable to patients, including those who experience the greatest disparities in CRC screening, including those with low incomes, those who are under- or uninsured, those with lower levels of education, and people from some racial and ethnic minority groups (e.g., Black, Hispanic, Alaska Native, or American Indian) (Levin et al., 2018; Mehta et al., 2016; Mousavinezhad et al., 2016). Future evaluations could assess which clinics are using different types of tests (e.g., FOBT, Cologuard), the reasons supporting their use, and the extent to which those clinics may be best supported to implement mailed FIT. This survey did not assess details of how often clinics mailed fecal screening tests and if they mailed tests to all patients overdue for CRC screening (opt-out strategy) or only to patients who requested kits or who agreed to receive kits prior to the mailing (opt-in strategy). Future studies should investigate these factors and strategies clinics have used to overcome known barriers to establishing the infrastructure required for coordinating mailed distribution and return. Finally, we did not evaluate mailed fecal testing costs; however, prior evaluations of the CRCCP suggest that automated patient reminders and financial incentives for support staff (e.g., medical assistants and office staff) enhance the cost-effectiveness of FIT and FOBT screening approaches, although this study did not differentiate between mailed fecal testing and tests distributed and returned at the clinic site (Tangka et al., 2020).

## Conclusions

Mailed fecal testing, an evidence-based approach that reduces disparities in CRC screening (Gupta et al., 2013), was widely implemented alongside multiple CPSTF-recommended EBIs in CHC and FQHC clinics participating in the CRCCP. As COVID-19 and future pandemics may continue to make in-person healthcare visits more difficult for some time, mailed fecal testing remains an important opportunity to reach populations disproportionately impacted by low CRC screening and poor CRC outcomes (Issaka & Somsouk, 2020; O’Connor et al., 2020). Our assessment of a group of clinics across the nation uncovered positive implementation practices among those implementing mailed fecal testing as well as opportunities for improvement. These opportunities include increasing the proportion of CHCs/FQHCs offering mailed screening; increasing the proportion that provide pre-paid return mail supplies with the screening kit; increasing the proportion of clinics monitoring both screening kit distribution and return; ensuring patients with positive tests can obtain colonoscopy; and increasing sustainability planning and support.

## Data Availability

The data analyzed in the current study are CDC program monitoring and evaluation data and are not publicly available.

## Declarations

### Etics Approval

This work was categorized as program evaluation and granted exemption by the University of Washington Institutional Review Board.
